# Effects of glycowithanolides on lipid peroxidation and lipofuscinogenesis in male reproductive organs of mice

**Published:** 2013-09

**Authors:** Madhuri Walvekar, Nilofar Shaikh, Priti Sarvalkar

**Affiliations:** *Department of Zoology, Shivaji University, Kolhapur, India.*

**Keywords:** Withania somnifera, Antioxidant, Lipid peroxidation, Cytoplasmic granules, Testes, Epididymis, Seminal vesicle.

## Abstract

**Background:** Glycowithanolides (Withaferin A), is one of the main withanolides active principle isolated from plant *Withania somnifera* and is claimed that it possess the aphrodisiac, sedative, rejuvenate and life prolonging properties.

**Objective: **In the present investigation, antioxidant activity of active principles of *Withania somnifera* was tested against D-galactose induced oxidative stress in mouse testes, epididymis and seminal vesicle.

**Materials and Methods:** For the present investigation Swiss male albino mice Mus musculus (Linn) were used. They were grouped in to control (I), D-galactose treated (II), protective (III) and curative groups (IV). Oxidative stress was induced in six month old mice by injecting a low dose of D-galactose. Antioxidant effect of plant extract was studied in testes, epididymis, and seminal vesicle of oxidative stressed mice on Lipid peroxidation (LPO) and fluorescence product.

**Results: **In the present study, both total as well as mitochondrial lipid peroxidation and fluorescence product in testes, epididymis and seminal vesicle were increased in D-galactose induced mice. After the treatment of glycowithanolides there was significantly decrease in total as well as mitochondrial lipid peroxidation and fluorescence product in protective and curative groups.

**Conclusion:** Our results indicate that *Withania somnifera* has a capability of preventing oxidative stress and also combating stress induced infertility.

## Introduction

Free radicals are highly reactive due to presence of unpaired electron. To nullify the adverse effect of the free radicals protective system is present. Super oxide dismutase (SOD), catalase (CAT), glutathione peroxidase (GPx) is the enzymatic antioxidants and non-enzymatic antioxidants are glutathione, vitamin E (Tocopherol), ascorbic acid centrophenoxine, and curotenoides (1-4). 

Cross linked product of oxidation damage are resistant to digestion by lysosomal enzyme. Lysosomes become unable to digest the phagocytosed material resulting into lipofuscinogenesis (5-7). Lipofuscinogenesis often exists in post mitotic cells of different animals (8, 9). Lipofuscin granules are of autofluroscent material which accumulates progressively with age in secondary lysosomes and linked with hydrolytic activity within lysosomes (10-14). In aging, mitochondria become enlarged, engulfed by lysosomes and contributes to formation of lipofuscin granules (15). Oxidative stress may result in unfavourable, physiological changes in the reproductive organs, including the epididymis and accessory glands (16). 

Damage localized to the epididymis may affect normal sperm maturation processes (17, 18). Therefore, oxidative stress coupled with aging correlates with decreased semen quality and causes infertility. The Reactive Oxygen Species (ROS) originating from spermatozoa are of significant patho-physiological importance in the etiology of male fertility (19-26). Mammalian sperm cells possesses highly specific lipidic composition and high content of polyunsaturated fatty acids and because of their capacity to generate ROS, human spermatozoa are very sensitive to oxidative stress (27, 19). The excessive production of ROS results in destruction of natural antioxidants capacity of reproductive tract (28). Stress is one of the important factors that induce infertility in normozoosprmic individuals (29).

*Withania somnifera *also called as ‘Ashwagandha’ belonging to Solanaceae family. It is one of the important herbs used in Ayurvedic medicine. It is used as general tonic to increase energy, improve all over health and longevity and prevent the diseases in athletes, the elderly, and during pregnancy. It may prevent tumour growth patient with cancer (30-32).

Glycowithanolides (Withaferin A) chemically characterized as 4b, 27-dihydroxy 5b-6b-epoxy-1 oxawitha-2, 24-dienolide, is one of the main withanolides active principles isolated from plant. *Withania somnifera* showed chemogenetic variation and so for three chemotype I, II, III had been reported (33). Therefore the aim of the present investigation was to study the protective effects of glycowithanolides on oxidative stress of male reproductive system to reduce infertility during aging. Oxidative stress was induced in adult male mice by injecting low dose of D-galactose (34).

## Materials and methods

**Plant material**

In this experimental study, fresh leaves of *Withania somnifera* were collected from Town Hall Garden Kolhapur. The plant was identified by Taxonomist from Botany Department, Shivaji University Kolhapur.

**Plant extraction**

Glycowithanolides were extracted from leaves of *Withania somnifera* plant as described by Bhattacharya *et al *(35). Fresh leaves of *Withania somnifera *were separated, washed with distilled water, blotted properly and kept for shade drying. Dried leaves were crushed, powdered and sieved. Then soaked in chloroform for 72 hrs to remove fatty material and separates withanolides. The solution was filtered and chloroform was evaporated by evaporator and thick paste was obtained. With the help of HPTLC the active principal glycowithanolides was confirmed. It was stored in glass bottle at 4^0^C and used as active ingredient for dose preparation.

**Animals**

Swiss albino male *Mus musculus *(Linn) of age six month old weighing 50-55 gr were used for present investigation. They were bred and reared in departmental animal house approved by Committee for the Purpose of Control and Supervision on Experiments on Animals. (CPCSEA/233) in separate cages under proper condition of light, temperature and humidity. They were supplied with Amrut mice feed (Pranav, Agro Industries, and Sangli) and water *ad libitum*. In total 20 animals were divided into 4 groups of 5 animals each. All animals were treated in accordance with the (CPCSEA), New Delhi, India.

1) Control group: Mice were injected subcutaneously with 0.5 ml sterile water/day/animal for 20 days.

2) D-galactose treated group: 5% D-galactose 0.5 ml/day/animal were injected subcutaneously for 20 days (34).

3) Protective group: Mice were injected subcutaneously with 0.5 ml of 5% D-galactose/day along with glycowithanolides 20 mg/kg body wt for 20 days. This dose was selected according to Bhattacharya *et al *(35).

4) Curative group: Mice were injected with 0.5 ml of 5% D-galactose for 20 days, and then to study the recovery, glycowithanolides were injected 20 mg/kg body wt for next 20 days. 

**Determination of total lipid peroxidation (LPO)**

After the completion of doses the animals were sacrificed by cervical dislocation, Testes, epididymis and seminal vesicle were dissected out, blotted and weighed. The tissues were homogenized in reaction mixture (2 mg/ml) containing 75mM phosphate buffer (pH= 7.04), 1 mM ascorbic acids and 1mM ferric chloride with 20% Trichloroacetic acid (TCA) and 0.67% Thiobarbituric acid (TBA).The mixture were heated in boiling water bath. The Thiobarbituric acid reacting substance TBARS in the form MDA was measured on spectrophotometer (Miltons Roy company) at 532 nm.

**Determination of mitochondrial lipid peroxidation**

For the mitochondrial fraction tissue was homogenized in 0.25 M sucrose and 1mM EDTA (2 mg/ml) and centrifugation was carried out at 3000 rpm for 10 min at 4^o^C (Cooling microfuge, Remi). The supernatant were again centrifuged at 10,000 rpm for 10 min at 4^o^C. The supernatant thus obtained were discarded, the pellete were resuspended in 0.2 ml 20% Triton X-100 and 0.8 ml distilled water and centrifuged at high speed 10.000 rpm for 10 min 4^o^C. 

The pellete obtained after high centrifugation were suspended in reaction mixture and used as sample for estimation of MDA in mitochondrial fraction. The total and mitochondrial lipid peroxidation was studied by Wills methods, in which thiobarbituric acid reactive substance (TBARS) i.e. Malondialdehyde (MDA) was measured in to form of red colored malondialdehyde- TBA spectrophotometer (Miltons Roy Co.) at 532 nm against blank (36). Lipid peroxidation was measured in the form of n mole MDA/mg wet tissue. 

**Measurement of** **fluorescence product**

Lipofuscinogenesis was studied Dillard and Tapple method (37). The testes, epididymis, and seminal vesicle were homogenized by using the mixture prepared earlier for lipid peroxidation. The extraction was carried out by addition of chloroform: methanol (2:1 v/v) to 0.5 ml of homogenized tissue sample. It was mixed well on vortex mixer and then 3ml of double distilled water was added and centrifuged at 300 g for 2 min. To 1ml of upper layer 0.1 ml of methanol was added and the fluorescence was measured on photoflurometer calibrated with Quinine sulphate. 

**Statistical Analysis**

The statistical analysis was performed using One way Analysis of Variance (ANOVA) followed by Tukey’s Post Hoc test. A value of p<0.01 was considered statistically significant. 

## Results

The lipid peroxidation (both total and mitochondrial lipid peroxidation) and fluorescence product in the testes, seminal vesicle and epididymis were increased in mice with D-galactose induced aging group (group II) as compared to control (group I) and this increase was highly significant (p<0.0001); while there was decrease in total as well as mitochondrial lipid peroxidation and fluorescence product in protective group (group III) and curative group (group IV) mice as compared to aging induced mice. In *Withania somnifera* treated groups significant results were observed in curative groups as compared to protective group (Table I, II and III).

**Table I:** Lipid peroxidation (n moles MDA /mg wet weight of tissue) and fluorescence product in testes of aging induced mice and effect of glycowithanolides on the same (mean±SD)

**Sr. No.**	**Groups (5)**	**Age of animals** **(in weeks)**	**Total lipid peroxidation**	**Statistical significance**	**Mitochondrial lipid peroxidation**	**Statistical significance**	**Fluorescence product**	**Statistical significance**
I	Control	25	11.51±0.0158		28.824±0.023		0.005828±0.000031	
II	D-galactose induced	25	28.843±0.019	1:2 p<0.0001	51.9204±0.021	1:2 p<0.0001	0.0176±0.000021	1:2 p<0.0001
III	Protective	27	23.0734±0.0024	2:3 p<0.0001	46.1504±0.021	2:3 p<0.0001	0.011736±0.000012	2:3 p<0.0001

Numbers in parenthesis denoted number of animals.

**Table II:** Lipid peroxidation (n moles MDA /mg wet weight of tissue) and fluorescence product in seminal vesicle of aging induced mice and effect of glycowithanolides on the same (mean ± SD)

**Sr. No.**	**Groups (5)**	**Age of animals** **(in weeks)**	**Total lipid peroxidation**	**Statistical significance**	**Mitochondrial lipid peroxidation**	**Statistical significance**	**Fluorescence product**	**Statistical significance**
I	Control	25	5.7654±0.0023		11.534±0.0032		0.003898±0.000019	
II	D-galactose induced	25	23.0734±0.0024	1:2 p<0.0001	40.382±0.0018	1:2 p<0.0001	0.01370±0.000021	1:2 p<0.0001
III	Protective	27	17.3028±0.0028	2:3 p<0.0001	28.8424±0.0023	2:3 p<0.0001	0.01173±0.000027	2:3 p<0.0001
IV	Curative	29	11.5342±0.0033	2:4 p<0.0001	23.0726±0.0024	2:4 p<0.0001	0.00584±0.00003	2:4 p<0.0001

Numbers in parenthesis denoted number of animals.

**Table III:** Lipid peroxidation (n moles MDA /mg wet weight of tissue) and fluorescence product in epididymis of aging induced mice and effect of glycowithanolides on the same (mean ± SD)

**Sr. No.**	**Groups (5)**	**Age of animals (in weeks)**	**Total lipid peroxidation**	**Statistical significance**	**Mitochondrial lipid peroxidation**	**Statistical significance**	**Fluorescence product**	**Statistical significance**
I	Control	25	5.766±0.0025		11.534±0.0032		0.00389±0.000019	
II	D-galactose induced	25	23.072±0.0029	1:2 p<0.0001	46.1504±0.0021	1:2 p<0.0001	0.00948±0.000025	1:2 p<0.0001
III	Protective	27	17.305±0.0016	2:3 p<0.0001	40.3812±0.0019	2:3 p<0.0001	0.00781±0.000019	2:3 p<0.0001
IV	Curative	29	11.536±0.0016	2:4 p<0.0001	23.0722±0.00029	2:4 p<0.0001	0.00584±0.000027	2:4 p<0.0001

Numbers in parenthesis denoted number of animals).

## Discussion

Free radicals oxidative stress has been implicated in the pathogenesis of a variety of diseases resulting usually from defective natural antioxidant defences. Potential antioxidants therapy should therefore, include either natural antioxidant enzymes or agents which are capable of augmenting the function of these oxidative free radical scavenging enzymes (38). 

In the present study the active principle glycowithanolides of *Withania somnifera* were found to decreases the lipid peroxidation and fluorescence product in testes, epididymis and seminal vesicle. D-galactose is a reducing sugar, which react non-enzymatically with amino group in protein, lipid, and nucleic acids and form advanced glycation end products (AGE_S_). AGE_S_ are responsible for production of free radicals thus they may accelerate the aging process (34). AGE_S_ accumulation in cell increases generation of ROS. These ROS cause the LPO of biomembrane through a chain reaction. The first step is initiation reaction, which begins by taking out “H” in unsaturated fatty acid by oxygen radicals. The second is the propagation and the final step is termination. 

The extent of LPO has often been determined by the thiobarbituratic acid (TBA) test, which has also been considered for the detection of malondialdehyde (MDA). A significant increase in (p<0.0001) MDA level from control to D-galactose induced mice in testes, epididymis and seminal vesicle indicates increases in LPO. In protective group this level was decreased significantly as compared to D-galactose induced group. While in curative group the LPO was still decreased and comes near to control group, indicating that instead of simultaneous treatment of glycowithanolides with D-galactose; the later treatment will be definitely beneficial. The increase in LPO leads to the damage of cell membrane. The membrane wastes are not digested properly due to insufficiency of lysosomal enzymes (39). These wastes get accumulated in the lysosomes and called lipofuscin granules (40). These lipofuscin granules are autofluroscent and that fluorescence we measured by Spectroflurometer (Spectroflurometer-ELICO). Increase in fluorescence in testes, epididymis and seminal vesicle in D-galactose treated mice indicates lysosomes are unable to digested wastes and increases in lipofuscin granules takes place. This fluorescence product decreased in curative and protective group indicating antioxidative effect of glycowithanolides.

The increase in LPO damage spermatozoon and increase male infertility, decrease sperm-egg interaction and reduces invivo fertility (27, 41, 42). Sukcharoen *et al* demonstrated the association of LPO with mid piece abnormality decreased sperm count, motility and loss of the capacity of the spermatozoan to undergo the acrosome reaction and fertilize (43). The present finding indicates that glycowithanolides offer protection against D-galactose induced oxidative stress in testes, epididymis and seminal vesicle.

## Conflict of interests

Authors do not have any conflict of interest.
